# Society Meetings

**Published:** 1897-08

**Authors:** 


### Society Meetings.

THE tenth annual meeting of the American Association of Obstetricians and Gynecologists will be held at the Cataract House, Niagara Falls, N. Y., Tuesday, Wednesday, Thursday and Friday, August 17, 18, 19 and 20, 1897.

The management of the Cataract House (which should be addressed on the subject) offers a special rate, American plan, to all guests who visit Niagara under the auspices of the association during that week. The Cataract will also provide a splendid room for the meeting.

The railways will sell return tickets to all who visit Niagara under our auspices for one-third the regular fare; *provided* the visitor obtains a certificate from the ticket agent at the starting point setting forth the transaction, which certificate must be signed at Niagara by the secretary of our association and there viséd by an agent of the Traffic Association. This agent will be in attendance on Wednesday and Thursday, ONLY.

OUTLINE PROGRAM.

The association will meet in executive session with closed doors on Tuesday, August 17th, at 9.30 o'clock A. M., for the election of new Fellows. The open session for the reading of papers will begin at 10 o'clock. Recess for luncheon at 1 o'clock P. M. Afternoon session at 3 o'clock and adjournment for the day at 5 o'clock.

The morning session will begin Wednesday at 10 o'clock for the reading of scientific papers. Recess at 1 o'clock. Afternoon session at 3 o'clock. Adjournment at 5 o'clock.

The morning session will begin Thursday at 10 o'clock to continue until 1 o'clock P. M., when recess for luncheon will be taken. The afternoon session will be called at 3 o'clock and at 5 o'clock adjournment of the scientific session for the day will take place.

At 6.30 P. M., Thursday, the executive session will convene for the election of officers and for such other business as may come before it.

At 8 o'clock P. M., Thursday, immediately after the executive session, the annual dinner will be given at the Cataract House.

Friday morning the closing session will begin at 9.30 o'clock. Final adjournment at 1 o'clock. A full attendance is specially requested at the final session.

It will be observed that there are to be no evening sessions for scientific work. The session appointed for Friday morning will make up for this, and the early adjournment each day (5.00 P. M.) will enable members to visit the several points of interest ; mornings, too, before 10 o'clock can be appropriated to this purpose. There is no more delightful place than Niagara in mid-August, and the business of the association can be conducted in the midst of the most fascinating surroundings, where both nature and art have conspired to excel in attractiveness. It is hoped that the Fellows in large numbers will improve this exceptional opportunity to combine pleasure and scientific work and unite to make this one of the most notable meetings yet held.

PAPERS PROMISED.

1. ............................President's address. James F. W. Ross, Toronto.

2. Pelvic inflammation : its causes and relations. A. P. Clarke, Cambridge.

3. Recent experiences with ventro-fixation. H. E. Hayd, Buffalo.

4. Surgical shock : its prevention and treatment. Walter B. Chase, Brooklyn.

5. Some remote sequelæ of the Baer operation. Rufus B. Hall, Cincinnati.

6. Puerperal diphtheria. H. W. Longyear, Detroit.

7. The prevention of pelvic disease. W. H. Humiston, Cleveland.

8. Appendicitis in relation to gynecology and pregnancy. John B. Deaver, Philadelphia.

9. Post-climacteric conditions that simulate advanced uterine cancer. M. Rosenwasser, Cleveland.

10. Ectopic gestation : a discussion of the changes occurring in the retained ovum, effused blood, and maternal tissues. L. H. Dunning, Indianapolis.

11. Placenta previa : with special reference to treatment. W. H. Wenning, Cincinnati.

12. Early diagnosis and treatment of cancer of the uterus. Wm. H. Myers, Fort Wayne.

13. Dynamic ileus following abdominal section. Frederick Blume, Allegheny.

14. What is the principal cause of puerperal sepsis? John Milton Duff, Pittsburg.

15. Incubation. John A. Lyons, Chicago.

16. Imperforate anus. John A. Lyons, Chicago.

17. Post-operative lesions and sequelæ: their extent, character and how to deal with them. Joseph Price, Philadelphia.

18. Reports of cases of reoperation following ventral fixation. Joseph Price, Philadelphia.

19. Two cases of intestinal obstruction: operation and recovery. William Wotkyns Seymour, Troy.

20. Technique of the dry method. Edwin Walker, Evansville.

21. The sequelæ of dead ligatures and sutures. George M. Hughes, Philadelphia.

22. Complete hysterectomy after injury during parturition and Cesarean section, with report of cases. Joseph H. Branham, Baltimore.

23. ..................................................

J. Henry Carstens, Detroit.

24. The operation itself in appendicitis. L. S. McMurtry, Louisville.

25. ..................................................

Charles A. L. Reed, Cincinnati.

26. Puerperal eclampsia: with special reference to treatment. William Warren Potter, Buffalo.

27. A case of Porro operation: recovery of mother and child. David Barrow, Lexington.

28. Tonic and spasmodic intestinal contractions: with report of cases. X. O. Werder, Pittsburg.

29. Fate of the ovaries in retroflexion and retroversion of the uterus. A. Goldspohn, Chicago.

30. Puerperal insanity. W. P. Manton, Detroit.

31. Cysts of the abdominal wall. Richard Douglas, Nashville.

32. Surgical treatment of puerperal sepsis: its possible prevention. Edwin Ricketts, Cincinnati.

33. Fifty cases illustrating personal experience in medical and surgical treatment of appendicitis. Geo. S. Peck, Youngstown.

34. Administration of phosphate of strychnia during gestation. W. B. Dorsett, St. Louis.

35. Masturbation in the female. B. Sherwood Dunn, Los Angeles.

36. Surgery of the kidney. J. B. Murphy, Chicago.

37. Treatment of puerperal endometritis by the Carossa method. Edward J. Ill, Newark.

38. Which is the preferable operation method of holding the uterus in position ? C. C. Frederick, Buffalo.

39. Limitations of the surgery of the liver. W. G. Macdonald, Albany.

One of the sessions will be devoted to the presentation of pathologic specimens and their histories, with discussion pertaining to the same. It is especially requested that photographs of these specimens be filed with the histories, from which engravings will be made for the transactions. It is the belief of the executive council that this may be made easily one of the most interesting sessions of the meeting.

No attempt is made to arrange papers in the order in which they are to be read. That will be done in the permanent program. Correspondence is pending concerning additional papers. All remaining titles must be offered before July 27th, when the permanent program goes to press.

Fellows and visitors are requested to register and file their railway certificates with the secretary promptly on arrival.

The Niagara Falls Academy of Medicine very generously offers to promote the success of the meeting in every way possible. A committee of the Academy will be in daily attendance to give information to the visitors, and especially to escort the ladies to places of interest or to direct them thither. This committee will be designated by a knot of blue ribbon on the left lapel, and it is requested that every one at the meeting will make free to call upon its members for information and service.

JAMES F. W. ROSS, *President.*

WILLIAM WARREN POTTER, *Secretary.*

---

THE American Microscopical Society will hold its next meeting at the Boody House, Toledo, on Thursday, Friday and Saturday, August 5, 6 and 7, 1897. The Toledo Microscopical Society has very cordially invited their brethren from other parts of the country to pay them a visit, and has promised to do all in its power to render that visit entertaining and instructive.

Those who attended the gathering at Pittsburg last year will recall the welcome tendered and the interest manifested by members in the Iron City and their friends, and it is hoped that all who can do so, will renew the experience by coming to Toledo in 1897.

The officers for the Toledo meeting are as follows: president, Professor E. W. Claypole, B. A., D. Sc., (Lond.) F. G. S., Buchtel College, Akron, O.; vice-president, C. C. Mellor, Pittsburg; secretary, William C. Krauss, M. D., Buffalo; treasurer, Magnus Pflaum, Pittsburg; executive committee, A. A. Young, M. D., Newark, N. Y.; Mrs. S. P. Gage, Ithaca, N. Y.; W. P. Manton, M. D., Detroit, Mich.

---

THE Buffalo Academy of Medicine held the following meeting in June not heretofore reported:

*Section on Surgery.*—Tuesday evening, July 29, 1897. Program: Some observations on the radical cure of hernia, by Dr. R. H. Harte, of Philadelphia, surgeon to the Pennsylvania hospital; exhibition of specimen, Dr. Eugene A. Smith; exhibition of cases, head injuries, Dr. Smith and Dr. Marshall Clinton.

Election of officers.

---

THE Medical Society of the County of Chautauqua held its regular annual meeting at the Hotel Atheneum, Chautauqua, N. Y., July 13, 1897, under the presidency of Dr. E. S. Rich. The annual election of officers resulted as follows: president, Dr. Morris H. Bemus, Jamestown; vice-president, Dr. V. M. Griswold, Fredonia; secretary-treasurer, Dr. Charles A. Ellis, Sherman; censors, T. D. Strong, Westfield; W. M. Bemus, Jamestown; J. Murphy, Sherman; delegates to the Medical Society of the State of New York, E. S. Rich, Kennedy, and C. A. Ellis, Sherman.

A photograph of the society was taken in the afternoon. The title of the president's address was Notes on puerperal eclampsia, in which he reported a large number of cases. Dr. W. W. Hotchkiss, of Jamestown, read a paper upon Tonsilitis; Dr. T. D. Strong, of Westfield, read upon Influenza. Dr. Lucien Howe, of Buffalo, presented a case of great interest to the society, of diphtheritic membrane of the eye-lid in a boy, which had existed for many months. At 8 P. M. Dr. R. R. Ross, superintendent of the Buffalo General Hospital, lectured upon the X-rays, illustrating with the apparatus as he proceeded, taking a photograph of a fractured femur at the close. The following physicians were present: Drs. M. H. Bemus, W. M. Bemus, E. M. Scofield, R. B. Parks, W. D. Wellman, J. H. Wiggins, W. W. Hotchkiss, Jamestown; E. S. Rich, Kennedy; E. A. Scofield, Bemus Point; T. D. Strong, W.

Stewart, Westfield ; N. G. Richmond, V. M. Griswold, Fredonia ; N. E. Beardsley, G. E. Blackham, Dunkirk ; H. J. Dean, A. E. Dean, Brocton ; W. C. Duke, Cassadaga ; R. M. Evarts, Silver Creek ; T. C. Wilson, Dewittville ; L. C. Green, Panama ; C. A. Ellis, G. D. Marsh, W. M. Haynes, J. Murphy, Sherman; R. R. Ross, Lucien Howe, Buffalo ; Eliza Mosher, Brooklyn ; W. S. Bainbridge, New York City ; J. W. Seaver, Yale University.

---

The Mississippi Valley Medical Association will hold its next meeting at Louisville, October 5, 6, 7 and 8, 1897. All railroads will offer reduced rates.

The president, Dr. Thos. Hunt Stucky, and the chairman of the committee of arrangements, Dr. H. Horace Grant, promise that the meeting will be the most successful in the history of the association, and this promise is warranted by the well-known hospitality of Louisville and Kentucky doctors.

Titles of papers should be sent to the secretary, Dr. H. W. Loeb, 3559 Olive street, St. Louis.

---

The Lake Erie Medical Society held its regular quarterly meeting at Angola, July 20, 1897. Fifteen members were present. Papers were read by Drs. Lake, of Gowanda, and Dods, of Fredonia. Dr. Beardsley, of Dunkirk, entertained the society.

